# Home delivered meals combined with clinical services may reduce fall risk among older adults: early findings from a pilot randomized controlled trial

**DOI:** 10.1093/geroni/igaf067

**Published:** 2025-06-26

**Authors:** Lisa A Juckett, Shivam Joshi, Govind Hariharan, Kali S Thomas, LifeCare Alliance

**Affiliations:** School of Health and Rehabilitation Sciences, The Ohio State University, Columbus, Ohio, United States; Center for Biostatistics, The Ohio State University Wexner Medical Center, Columbus, Ohio, United States; Coles College of Business, Kennesaw State University, Kennesaw, Georgia, United States; School of Nursing, Johns Hopkins University, Baltimore, Maryland, United States; LifeCare Alliance, Columbus, Ohio, United States

**Keywords:** Homebound, Nutrition, Occupational therapy, Registered dietitian, Home assessment

Translational Significance
Without innovative and effective strategies to mitigate fall risk, homebound older adults will remain vulnerable to falls, jeopardizing their safety and independence within their homes. Findings from this study suggest that integrating registered dietitian nutritionist and occupational therapy services with meal deliveries may contribute to fall risk reduction and support older adults in successfully aging within their preferred homes and communities. These results underscore the need for a fully powered follow-up trial to rigorously evaluate the true effect of tailored clinical services on fall prevention among recipients of home delivered meals.

## Background and objectives

Older adults who receive home delivered meals experience falls at a rate nearly double that of the general aging population.^1^ Though some research suggests that meals may help to reduce falls,^2^ fall prevention strategies among older home delivered meal recipients remain understudied. However, given that 80% of meal recipients have at least one mobility impairment^3^ and 90% are at risk of malnutrition,^4^ a well-established fall risk factor,^5^ effective approaches to manage fall risk among this population are needed. Accordingly, the purpose of this study was to determine the preliminary effects of tailored clinical services—that is, registered dietitian nutritionist (RDN) and occupational therapy (OT) services—on fall risk among homebound older adults receiving home delivered meals.

## Research design and methods

### Design and setting

This four-arm pilot randomized controlled trial (RCT) recruited participants aged 60+ years who received home delivered meals from one large, social service agency in the Midwest United States. Eligible participants had to have at least one fall risk factor (eg, prior falls, fear of falling, and mobility challenges) and had a self-reported diagnosis of diabetes or cardiovascular disease (ie, diet-related conditions that increase the risk of falling). All study activities were conducted in accordance with guidance from the Consolidated Standards of Reporting Trials (CONSORT) statement for randomized pilot and feasibility trials.^6^ Please see study design details at clinicaltrials.gov (NCT06059404).

### Procedures

Once consented, participants completed baseline in-person surveys with a trained member of our team and were randomi­zed into one of our four study arms (described later). After receiving meal services for 12 weeks, our team readministered follow-up surveys to participants in their homes to determine changes in perceived fall risk over time.

### Intervention arms

Participants randomized into Arm 1 (meals only) received 7 to 14 frozen meals that were delivered once a week for 12 weeks. Participants were able to choose their meals each week from a list of 40 options. In Arm 2 (meals + RDN), participants received frozen meals that were selected with the guidance of a RDN who completed an initial and follow-up phone call encounter with each participant. Arm 3 (meals + OT) participants selected their frozen meals and received an in-home OT evaluation and fall prevention recommendations. Arm 4 (meals + RDN + OT) participants received the combination of frozen meals as well as RDN and OT services.

### Outcome measures

Our primary outcome was perceived fall risk as measured by the Short Falls-Efficacy Scale-International (FES-I), which is a seven-item questionnaire that evaluates participants’ level of concern with falling when performing daily tasks. The scale has excellent test-retest reliability (Cronbach’s alpha = 0.92), with higher scores (max score = 28) indicative of greater concerns with falling.^7^ Demographic variables were also collected via self-report over the telephone from all participants.

### Analysis

Multivariable analysis was conducted to assess changes in perceived fall risk among arms. Effect sizes were calculated using marginal estimates from a repeated-measures, mixed effects linear regression model.^8^ To help interpret the magnitude of preliminary intervention effects, we also calculated Cohen’s *d* for each study arm.

## Results

We enrolled a total of 56 participants over the course of 6 months. There were slightly more women who enrolled than men (50% vs 46.4%; 3.6% did not self-disclose); 25.0% were between the ages of 65 to 69 years of age; 58.9% of our sample was White, and half of our participants lived alone. There were no statistically significant differences in our sample’s baseline characteristics, indicating our randomization scheme was sufficient for sustaining balance across all four arms.

Baseline and 3-month follow-up FES-I scores are presented in Table 1. As all study arms demonstrated statistically signifi­cant changes in FES-I scores over time, effect sizes were calculated to evaluate the magnitude of these differences. Results indicated moderate effect sizes across all intervention arms, with the largest effect observed in the Meals + RDN + OT group (*d *= −0.74) and the smallest in the Meals only group (*d *= −0.56). The Meals + RDN (*d *= −0.64) and Meals + OT (*d *= −0.63) arms exhibited comparable effect sizes.

**Table 1.** Mean differences in baseline and 3-month follow-up FES-I scores

	Pre (*M*; *SD*)	Post (*M*; *SD*)	Mean difference	*P*	Cohen’s *d*
Meals only	17.08 (1.17)	12.13 (1.49)	−4.95 (1.89)	.0131^a^	−0.56
Meals + RDN	18.77 (1.17)	13.11 (1.40)	−5.66 (1.82)	.0039^a^	−0.64
Meals + OT	16.93 (1.12)	11.80 (1.33)	−5.13 (1.74)	.0058^a^	−0.63
Meals + RDN + OT	17.92 (1.17)	11.91 (1.27)	−6.01 (1.72)	.0014^a^	−0.74

Abbreviations: FES-I = Short Falls-Efficacy Scale-International; OT = occupational therapy services; pre = baseline; post = 3-month follow-up; RDN = registered dietitian nutritionist services.

a Statistically significant (α = .05). Meals only (*n* = 14); meals + RDN (*n* = 14); meals + OT (*n* = 14); meals + RDN + OT (*n* = 14).

## Discussion and implications

Our findings indicate that the delivery of meals combined with tailored clinical services may lead to a moderate reduction in older adults’ perceived concern with falling, with the greatest reductions among those who receive the *combination* of RDN and OT services. These results offer promising solutions for reducing falls among home delivered meal recipients who exhibit a high prevalence of fall risk factors.^3^ Given that this homebound population faces marked difficulties accessing clinical services to address fall risk, positioning RDN and OT services within home delivered meal agencies reduces access barriers for homebound older adults who have been historically underserved by the healthcare system.^9^

Although this study represents an encouraging approach to fall prevention, several limitations should be acknowledged. As a pilot study, it was not sufficiently powered to detect the true effect of clinical services on FES-I scores. However, our primary objective was not to draw definitive statistical conclusions but rather to estimate effect sizes and assess the feasibility of study procedures in preparation for a fully powered ­follow-up trial. Additionally, this study was conducted in collaboration with a single home delivered meal agency, which may limit the generalizability of findings to meal recipients nationwide. Lastly, the FES-I measured *perceived* fall risk rather than participants’ *actual* rates of falling. Given that community-dwelling older adults often underreport their fall history,^10^ a follow-on study after our full trial may be warranted using administrative data (eg, Medicare claims data) to understand clinical services’ effect on actual falls. Nonetheless, given the innovative nature of this work, the study makes a valuable contribution to the field of aging and enhances the existing literature on fall prevention among high-risk older adults.

## Data Availability

The datasets and analytic methods used during the described study are available from the corresponding author on reasonable request. Study activities were preregistered with clinicaltrials.gov (NCT06059404).
